# HIV-1 Efficient Entry in Inner Foreskin Is Mediated by Elevated CCL5/RANTES that Recruits T Cells and Fuels Conjugate Formation with Langerhans Cells

**DOI:** 10.1371/journal.ppat.1002100

**Published:** 2011-06-30

**Authors:** Zhicheng Zhou, Nicolas Barry de Longchamps, Alain Schmitt, Marc Zerbib, Marie-Cécile Vacher-Lavenu, Morgane Bomsel, Yonatan Ganor

**Affiliations:** 1 Mucosal Entry of HIV-1 and Mucosal Immunity, Cell Biology and Host Pathogen Interactions Department, Cochin Institute, CNRS (UMR 8104), Paris, France; 2 INSERM, U1016, Paris, France; 3 Université Paris Descartes, Paris, France; 4 Electron Microscopy Platform, Cochin Institute, CNRS (UMR 8104), Paris, France; 5 Urology Service, GH Cochin-St Vincent de Paul, Paris, France; 6 Department of Pathology, GH Cochin-St Vincent de Paul, Paris, France; Emory University, United States of America

## Abstract

Male circumcision reduces acquisition of HIV-1 by 60%. Hence, the foreskin is an HIV-1 entry portal during sexual transmission. We recently reported that efficient HIV-1 transmission occurs following 1 h of polarized exposure of the inner, but not outer, foreskin to HIV-1-infected cells, but not to cell-free virus. At this early time point, Langerhans cells (LCs) and T-cells within the inner foreskin epidermis are the first cells targeted by the virus. To gain in-depth insight into the molecular mechanisms governing inner foreskin HIV-1 entry, foreskin explants were inoculated with HIV-1-infeceted cells for 4 h. The chemokine/cytokine milieu secreted by the foreskin tissue, and resulting modifications in density and spatial distribution of T-cells and LCs, were then investigated. Our studies show that in the inner foreskin, inoculation with HIV-1-infected cells induces increased CCL5/RANTES (1.63-fold) and decreased CCL20/MIP-3-alpha (0.62-fold) secretion. Elevated CCL5/RANTES mediates recruitment of T-cells from the dermis into the epidermis, which is blocked by a neutralizing CCL5/RANTES Ab. In parallel, HIV-1-infected cells mediate a bi-phasic modification in the spatial distribution of epidermal LCs: attraction to the apical surface at 1 h, followed by migration back towards the basement membrane later on at 4 h, in correlation with reduced CCL20/MIP-3-alpha at this time point. T-cell recruitment fuels the continuous formation of LC-T-cell conjugates, permitting the transfer of HIV-1 captured by LCs. Together, these results reveal that HIV-1 induces a dynamic process of immune cells relocation in the inner foreskin that is associated with specific chemokines secretion, which favors efficient HIV-1 entry at this site.

## Author Summary

Circumcision reduces HIV-1 acquisition in men, suggesting that the foreskin is an HIV-1 entry site. We previously showed that two types of immune cells in the foreskin epidermis are the first ones targeted by HIV-1. Hence, Langerhans cells (LCs) positioned in proximity to the external surface rapidly capture incoming HIV-1. The internalized virus is then transferred to T-cells positioned deeper within the epidermis. Herein, we studied the molecular mechanisms affecting the movement of these motile cells by testing whether HIV-1 alters the secretion of foreskin chemokines (i.e. molecules that influence cell migration). Our results show that HIV-1 increased the secretion of CCL5/RANTES, a potent T-cell chemokine, which mediated T-cell recruitment into the epidermis. In parallel, HIV-1 decreased the secretion of CCL20/MIP-3 alpha, a potent LC chemokine, enabling LCs to travel deeper into the tissue. The two cell types then ‘meet’ to form close contacts that permit the transfer of the virus from LCs to T-cells within the epidermis. Together, these results reveal that HIV-1 modifies foreskin chemokines secretion and the subsequent relocation of its initial immune target cells. Therefore, blocking the responsiveness to these chemokines clinically may limit the local spread of HIV-1 within the foreskin.

## Introduction

According to an updated report on the global AIDS epidemic (see www.unaids.org), 15 million men are currently infected with HIV-1 worldwide. HIV-1 infection in men has recently gained extensive scientific and public attention following reports of three clinical trials, which clearly demonstrated that male circumcision provides 60% protection from HIV-1 acquisition [1]–[3]. These reports confirmed a multitude of previous similar epidemiological observations [4], and suggest altogether an important role of the male foreskin as an entry portal for HIV-1.

The male foreskin is a stratified epithelium, made of multiple layers of keratin-forming epithelial cells (i.e. epidermis) positioned on top of a connective tissue made of collagen-producing fibroblasts (i.e. dermis) [5]. The foreskin consists of two different aspects, outer and inner, which are easily distinguished by the relative decrease in melanocytes in the inner foreskin [6]. While some studies, including ours, have reported that the degree of keratinization of the outer foreskin is higher than that of the inner [7]–[10], other studies reached opposite conclusions [11] or reported no difference in the degree of foreskin keratinization [12]. Hence, a standardized method to evaluate keratin thickness is required, in order to determine morphologically the difference in keratinization between the outer and inner foreskins, which may provide a protective barrier against HIV-1 entry [7], [9], [10], [13].

Both foreskin epidermis and dermis also contain various immune cells, such as Langerhans cells (LCs), T-cells, dendritic cells (DCs) and macrophages [6]–[8], [10], [14]–[16]. These immune cells may serve as potential targets for HIV-1 due to their expression of CD4/CCR5, the principal receptors for HIV-1 [6]–[8], [10], [14], [16], [17], as well as alternative HIV-1 attachment receptors, such as the C-type lectins langerin on LCs and Dendritic Cell-Specific Intercellular adhesion molecule-3-Grabbing Non-integrin (DC-SIGN) on DCs [10], [15], [18]–[22]. Two previous studies showed that the foreskin is susceptible to entry of high doses of cell-free HIV-1 at time points of >24 h [7], [16]. However, until recently, the exact mechanisms responsible for HIV-1 entry into the foreskin, especially during the very first hours following exposure of the foreskin to HIV-1, remained to a large extent unknown.

To describe the initial events of HIV-1 foreskin entry, we recently developed two novel and complementary models of the adult human foreskin epithelium, namely *ex-vivo* polarized inner and outer foreskin explants, and immuno-competent stratified foreskin epithelia reconstructed *in-vitro* from isolated primary inner or outer foreskin cells [10]. In these models, efficient HIV-1 transmission occurs following 1 h of polarized exposure of the inner, but not outer, foreskin to mononuclear cells highly infected with HIV-1, but not to cell-free virus [10]. Hence, within the inner foreskin, in a first step, HIV-1-infected cells form viral synapses with apical foreskin epithelial cells, leading to polarized budding of HIV-1; In a second step, HIV-1 is rapidly internalized by epidermal LCs and remains intact within their cytoplasm; In a third step, LCs transfer HIV-1 to T-cells within the foreskin epidermis, across conjugates formed between these two cell types [10]. Together, these studies identified the foreskin region permissive to HIV-1 (i.e. the inner foreskin), the conditions allowing for efficient viral entry (i.e. virus originating from infected cells), and the initial cells targeted by HIV-1 (i.e. LCs and T-cells).

In the present study, we now explore the molecular mechanisms responsible for potent inner foreskin HIV-1 entry. Thus, our recently developed polarized foreskin explants were inoculated with HIV-1-infeceted or non-infected cells for 4 h. Following viral exposure, the chemokine/cytokine milieu secreted by the foreskin tissue was examined, as well as any related changes in the density and spatial distribution of T-cells and LCs. The involvement of selective chemokines in these processes was confirmed using relevant blocking Abs. The results presented herein demonstrate specific regulation of chemokines secretion within the inner foreskin, correlating with a dynamic process of HIV-1 target cell migration, which may govern the efficient entry of HIV-1 at this site.

## Results

### HIV-1-infected cells affect specific chemokines secretion by the inner foreskin

T-cells and LCs are the first cells targeted by HIV-1 during early viral exposure of the inner foreskin [10]. The motility of these migratory cells dependents on environmental cues, such as chemokines and cytokines secreted by the tissue. To first screen for potential chemokines and cytokines secreted by the foreskin, inner foreskin explants were inoculated in a polarized manner for 4 h at 37°c with either non-infected or HIV-1-infected peripheral blood mononuclear cells (PBMCs). Explants were then washed and further incubated in culture medium in a non-polarized manner overnight at 37°c, to permit the secretion of high/detectable chemokines/cytokines levels. Quantitative multiplex bead immunoassay and flow cytometry or specific ELISA was then used to measure the levels of twelve chemokines/cytokines.

Exposure of inner foreskin explants to non-infected PBMCs (serving as negative control) resulted in different levels of chemokines/cytokines secretion: no secretion of IL-23, IL-17A, IL-10 and IL-1 beta; low levels of IFN gamma (<20 pg/ml); moderate levels of CCL3/MIP-1 alpha, CCL20/MIP-3 alpha, and CCL5/RANTES (100–1000 pg/ml); and high levels of CCL2/MCP-1, CXCL8/IL-8, IL-6 and CXCL10/IP-10 (2500–18500 pg/ml).

**Figure 1:**
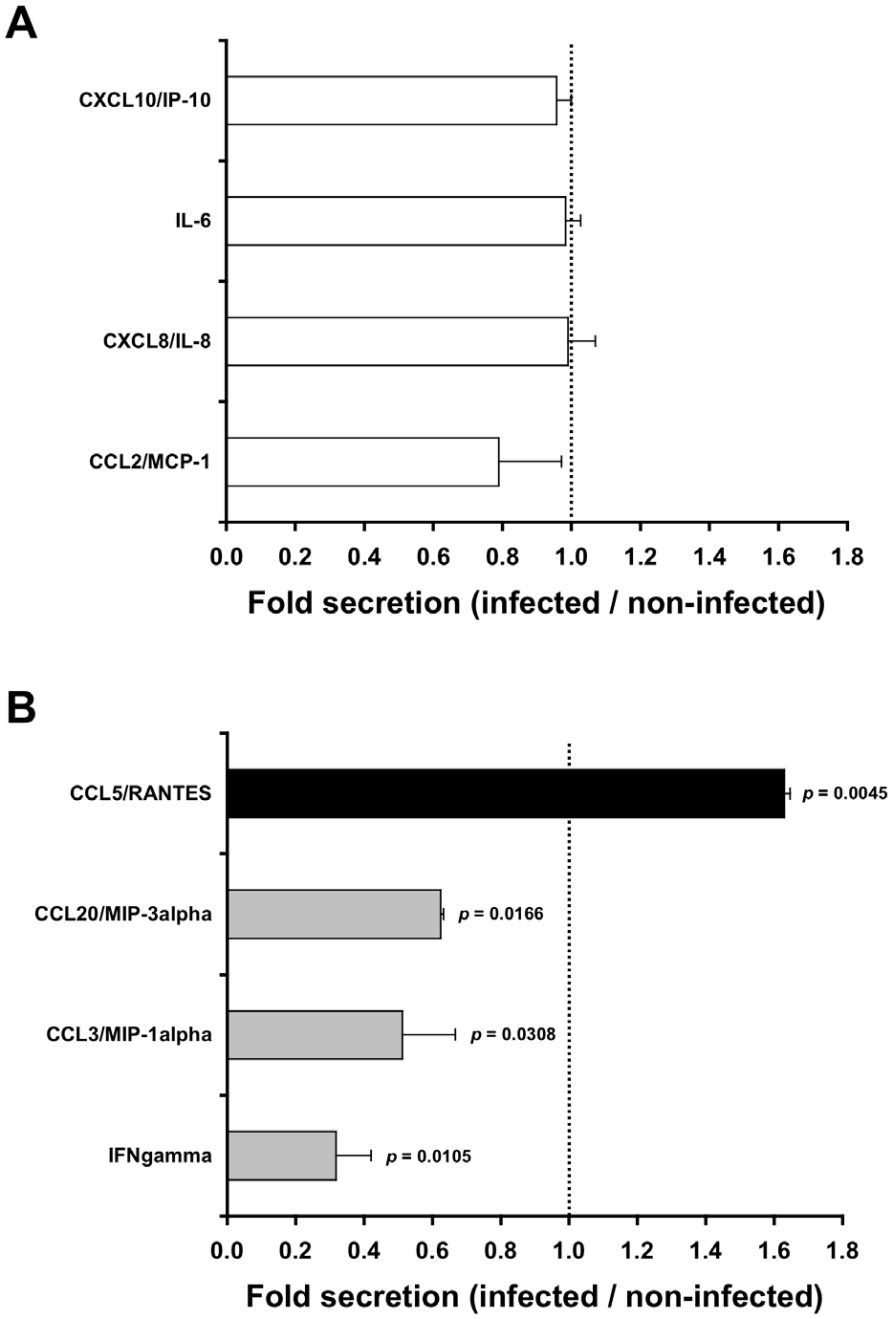
HIV-1-infected cells affect secretion of chemokines by the inner foreskin. Inner foreskin explants were inoculated in a polarized manner for 4 h with either non-infected or HIV-1-infected PBMCs, washed, and further incubated in medium in a non-polarized manner. The levels of twelve chemokines and cytokines were measured the next day in the culture supernatants, using a custom multiplex bead immunoassay and quantified by flow cytometry or specific ELISA. Shown are mean folds±SEM secretion of each analyte (derived from two independent explants), calculated as [secretion after exposure to HIV-1-infected PBMCs/secretion after exposure to non-infected PBMCs], for either chemokines/cytokines not altered (A) or altered (B) following exposure to HIV-1-infected cells. In (B), p = 0.0105, 0.0308, 0.0166, 0.0045 for INF gamma, CCL3/MIP-1 alpha, CCL20/MIP-3 alpha and CCL5/RANTES, respectively, Student's t-test, n = 2.

Next, to identify chemokines/cytokines whose secretion was specifically affected by the interaction of HIV-1 with the tissue, the measured levels following inoculation with HIV-1-infected PBMCs were normalized to that following inoculation with non-infected PBMCs. This enables the calculation of folds increase/decrease secretion. Among the chemokines/cytokines secreted by the inner foreskin, the levels of the highly secreted ones (i.e. CCL2/MCP-1, CXCL8/IL-8, IL-6 and CXCL10/IP-10) were not changed by the presence of HIV-1 (Fig. 1A). In contrast, following inoculation with HIV-1-infected PBMCs, the chemokines/cytokines secreted at low/moderate levels were either decreased, including IFN gamma, CCL3/MIP-1 alpha and CCL20/MIP-3 alpha (Fig. 1B, grey bars; mean actual values were 13.8, 590.5 and 443.0 pg/ml following inoculation with non-infected PBMCs and 4.0, 310.1 and 276.9 pg/ml following inoculation with HIV-1-infected PBMCs, respectively; n = 2), or increased, including CCL5/RANTES (Fig. 1B, black bar; mean actual values were 580.3 pg/ml following inoculation with non-infected PBMCs and 953.6 pg/ml following inoculation with HIV-1-infected PBMCs, respectively; n = 2). Of note, in outer foreskin explants (from the same individuals and tested in parallel experiments), CCL5/RANTES was also secreted at moderate levels, but remained unchanged following exposure to HIV-1-infected PBMCs (mean actual values were 291.5 pg/ml following inoculation with non-infected PBMCs and 354.3 pg/ml following inoculation with HIV-1-infected PBMCs, respectively; n = 2, p = 0.3451, Student's t-test).

To further examine whether these alterations in chemokines secretion were already detectable at the end of the infection period, inner foreskin explants were inoculated in a polarized manner for 4 h at 37°c with either non-infected or HIV-1-infected PBMCs, and the apical and basal supernatant fractions were collected immediately. A second array consisting of glass slides spotted with capture Abs to either CCL5/RANTES or CCL20/MIP-3 alpha, as well as specific CCL20/MIP-3 alpha ELISA, were then used to evaluate the early-secreted levels of both chemokines. The obtained values (i.e. arbitrary relative fluorescent intensities using slides or pg/ml using ELISA) were translated to folds increase/decrease secretion.

Although the secretion levels of both chemokines at this early time point were lower and variable among foreskin tissues from different individuals, inoculation with HIV-1-infected PBMCs modified the secretion of CCL5/RANTES and CCL20/MIP-3 alpha in inner foreskin explants in a similar fashion to that observed at later time points. Hence, compared to non-infected PBMCs, exposure to HIV-1-infected PBMCs resulted in an increase in early CCL5/RANTES that was secreted only into the apical supernatant fraction (mean fold increase±SEM of 1.397±0.045 from n = 3 explants; p = 0.0419, Student's t-test). In parallel, exposure to HIV-1-infected PBMCs resulted in a decrease in early CCL20/MIP-3 alpha that was secreted both apically and basally (mean folds increase±SEM of 0.521±0.183 apical and 0.558±0.163 basal from n = 3 explants; p = 0.0345 and 0.0411, respectively, Student's t-test).

Finally, immunohistochemical staining of inner foreskin explants for CCL5/RANTES expression showed that this chemokine was expressed by foreskin keratinocytes. Unfortunately, despite using several CCL5/RANTES Abs, the detected signals were low and similar in all conditions, thus not permitting for quantitative evaluation of the differences in CCL5/RANTES levels following exposure to either non-infected or HIV-1-infected PBMCs. Immunohistochemistry appears therefore not sensitive enough to evaluate these differences, which were in contrast observed by the more sensitive and quantitative methods we used (i.e. ELISA and array slide) at this early time point.

These results show that HIV-1-infected PBMCs affect rapidly the secretion of specific chemokines by the inner foreskin that persists at later time points following viral exposure.

### HIV-1-infected cells induce T-cells recruitment into the inner foreskin epidermis

CCL5/RANTES exerts chemotactic activity on T-cells [23], [24]. In the foreskin, the majority of T-cells are located within the dermis, and a minority of T-cells are integrated within the epidermis [6]–[8], [10], [14]–[16]. As secretion of CCL5/RANTES increased in the inner foreskin epidermis upon early exposure to HIV-1-infected PBMCs (see above), we next investigated possible changes in T-cell density in inner foreskin following viral exposure. Inner and outer foreskin explants were inoculated comparatively with HIV-1-infected or non-infected PBMCs, and 4 h later the explants were fixed, processed for immunohistochemistry, and stained for CD3 expression. The number of the positively stained CD3+ T-cells was then quantified in both epidermis and dermis.

**Figure 2:**
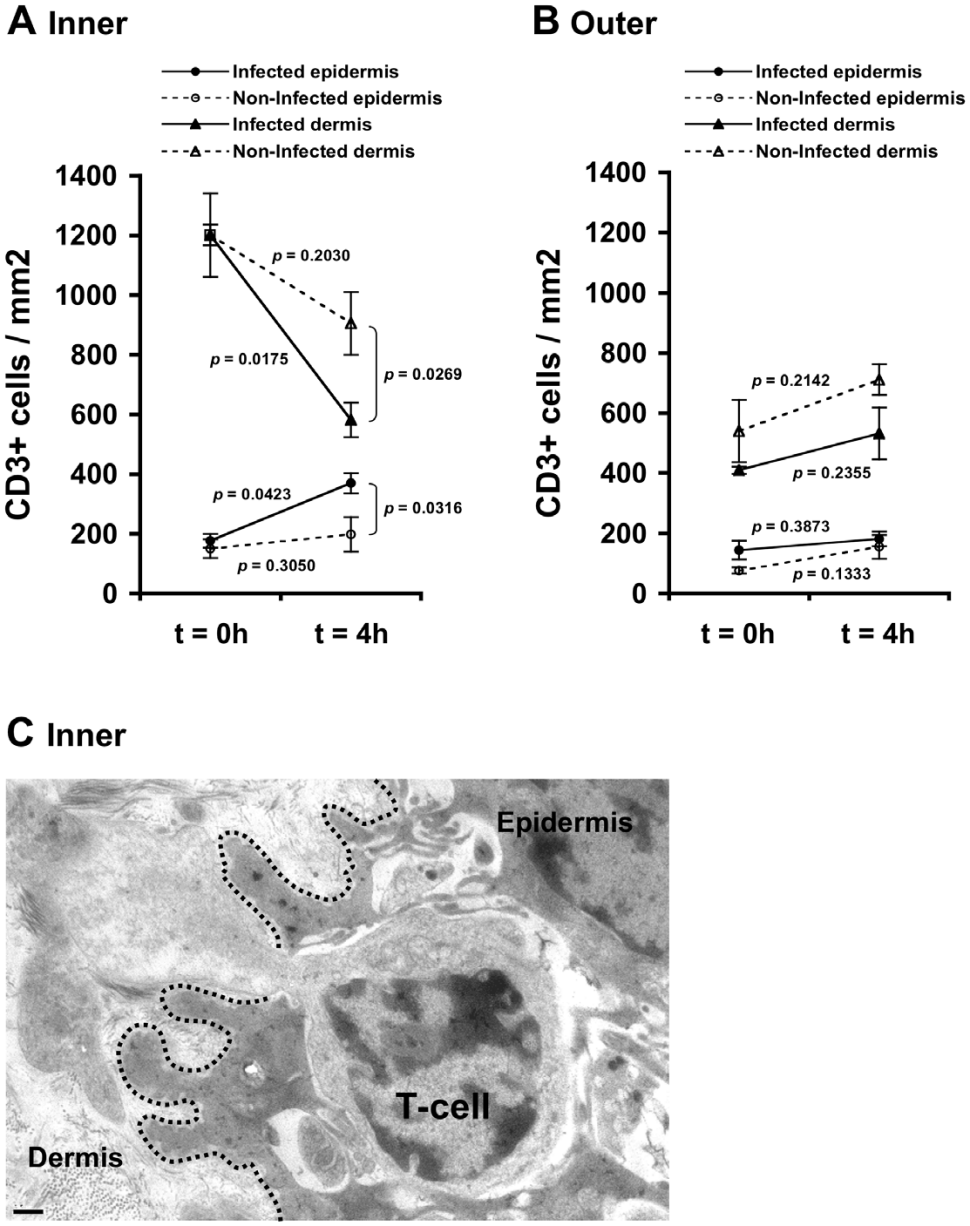
HIV-1-infected cells induce recruitment of T-cells into the epidermis. Parallel inner (A) and outer (B) foreskin explants were exposed for 4 h to either HIV-1-infected (solid lines) or non-infected (broken lines) PBMCs. Explants were then stained with anti-CD3 Ab and visualized with DAB peroxidase substrate. Shown are means±SEM CD3+ cell densities (cells/mm^2^) in either epidermis or dermis derived from three independent explants. Cells were counted in a minimum of 10 fields/section in each experiment. In (A): p = 0.0423 and 0.0175, 4 h vs. 0 h for infected epidermis and dermis, respectively; p = 0.0316 and 0.0269, HIV-1-infected vs. non-infected at 4 h for epidermis and dermis, respectively; Student's t-test, n = 3. (C) Electron micrograph of inner foreskin explant exposed for 4 h to HIV-1-infected PBMCs. Shown is a T-cell, identified by its typical morphology and size/shape of nucleus, migrating into the epidermis across a disrupted basement membrane (non-continuous black dotted line). The rear of the cell is still localized in the dermis. Scale bar = 0.5 µm.

Following 4 h inoculation with HIV-1-infected PBMCs, the density of epidermal T-cells in the inner foreskin significantly doubled (from 177±31 to 370±58; mean±SEM CD3+ cells/mm^2^ epidermis from n = 3 experiments; p = 0.0423, Student's t-test; Fig. 2A). In contrast, inoculation with non-infected PBMCs did not significantly change the density of epidermal T-cells (from 151±23 to 199±34; mean±SEM CD3+ cells/mm^2^ epidermis from n = 3 experiments; p = 0.3050, Student's t-test; Fig. 2A). The difference between the densities of epidermal T-cells following exposure to HIV-1-infected compared to non-infected PBMCs at 4 h was significant (p = 0.0316, Student's t-test). The increase in T-cell density in the inner foreskin epidermis following exposure to HIV-1-infected PBMCs was correlated with a significant decrease in their density within the inner foreskin dermis (from 1201±140 to 582±58; mean±SEM CD3+ cells/mm^2^ dermis from n = 3 experiments; p = 0.0175, Student's t-test; Fig. 2A). Although the density of dermal T-cells decreased following inoculation with non-infected PBMCs, the changes were not statistically significant (p = 0.2030, Student's t-test; Fig. 2A). The difference between the densities of dermal T-cells following exposure to HIV-1-infected compared to non-infected PBMCs at 4 h was significant (p = 0.0269, Student's t-test).

T-cell recruitment described herein is not a result of tissue entry of the input PBMCs. Indeed, fluorescently loaded HIV-1-infected input cells were never detected within the epithelium or the basal compartment after their inoculation at the apical surface (data not shown).

To gain further insight as to the identity of T-cells that entered the epidermis, we performed additional double immunohistochemical staining experiments of inner foreskin explants and examined CD4 and CD8 expression on CD3+ T-cells. At 4 h, the ratio of epidermal CD8 over CD4 T-cells was 8.9±1.3 following inoculation with non-infected PBMCs. Following exposure to HIV-1-infected PBMCs, the epidermal densities of both CD4+ and CD8+ cells increased, but the CD8 over CD4 ratio decreased to 3.7±0.6 (p = 0.0114, Student's t-test, n = 3), suggesting that HIV-1-infected PBMCs induced preferential recruitment of CD4+ cells into the epidermis.

In agreement with these findings, the above-mentioned changes corresponded with migration of T-cells from the dermal into the epidermal compartments across a disrupted basement membrane, as observed at the ultra-structural level in inner foreskin explants following 4 h exposed to HIV-1-infected PBMCs (Fig. 2C).

In contrast, no statistically significant changes were observed in the density of epidermal/dermal T-cells in parallel outer foreskin explants exposed to either HIV-1-infected or non-infected PBMCs (p = 0.3873, 0.1333, 0.2355 and 0.2142 following exposure to HIV-1-infected or non-infected PBMCs for either epidermis or dermis, respectively; Student's t-test; Fig. 2B). T-cells translocation from the dermis into the epidermis was not observed at the ultra-structural level in outer foreskin explants following 4 h exposure to HIV-1-infected PBMCs.

These results suggest that HIV-1 originating from infected cells recruits T-cells from the dermis into the epidermis in inner, but not outer, foreskin.

### CCL5/RANTES mediates T-cell recruitment into the inner foreskin epidermis

To investigate whether the increase in CCL5/RANTES secretion by the inner foreskin epidermis, detected after 4 h viral exposure, is responsible for the observed T-cell recruitment (Fig. 2), a neutralizing Ab to CCL5/RANTES was used. Hence, inner foreskin explants from additional individuals were exposed for 4 h at 37°c to HIV-1-infected PBMCs either: 1) alone; 2) in the presence of a control isotype Ab; 3) or in the presence of a CCL5/RANTES neutralizing Ab. Exposure to medium or non-infected PBMCs served as controls. Following infection, epidermal single-cell suspensions were prepared, stained for CD3 expression and examined by flow cytometry.

**Figure 3:**
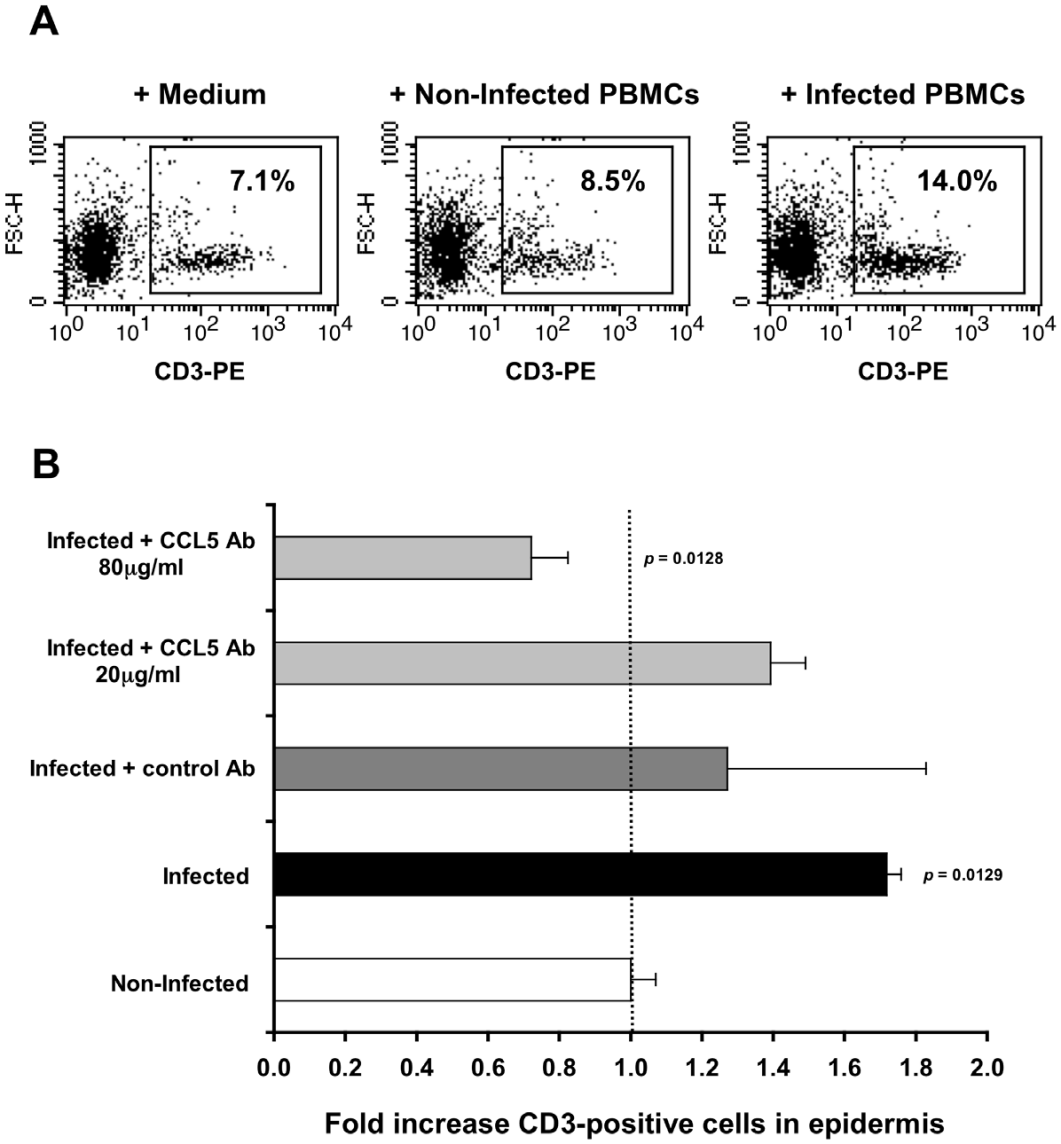
CCL5/RANTES mediates T-cell recruitment into the epidermis. (A) Representative FACS profiles out of two independent experiments, showing epidermal single-cell suspensions derived from inner foreskin explants exposed for 4 h to either medium alone (left), non-infected PBMCs (middle), or HIV-1-infected PBMCs (right). Cells were stained with PE-conjugated anti-human CD3 mAb and numbers represent the percentages of CD3+ cells in the framed windows (determined based on the non-specific staining of a matched isotype control Ab). (B) Inner foreskin explants were exposed for 4 h to either non-infected PBMCs (white bar) or HIV-1-infected PBMCs: alone (black bar); in the presence of 40 µg/ml control goat IgG Ab (dark grey bar); in the presence of 20 or 80 µg/ml neutralizing goat Ab to CCL5/RANTES (light grey bars). Following infection, epidermal single-cell suspensions were prepared and stained for CD3 expression as described above. Shown are mean folds increase±SEM of the percentages of CD3+ cells, normalized against that following exposure to non-infected PBMCs and derived from two independent explants. p = 0.0129 infected vs. non-infected and p = 0.0128 infected+CCL5 Ab 80 µg/ml vs. infected; Student's t-test.

In agreement with our CD3+ cell counting by immunohistochemistry (Fig. 2A), inoculation with HIV-1-infected PBMCs resulted in a higher percentage of CD3+ cells in cell suspensions of inner foreskin epidermis, compared to inoculation with either medium of non-infected PBMCs (Fig. 3A, B). Pre-treatment of inner foreskin explants with a control isotype Ab did not affect the observed increase in CD3+ cells upon exposure to HIV-1-infected cells (Fig. 3B, dark grey bar). In sharp contrast, a neutralizing Ab to CCR5/RANTES completely abrogated T-cell recruitment into the epidermis (Fig. 3B, light grey bar, 80 µg/ml Ab).

This set of results demonstrates that the recruitment of T-cells into the inner foreskin epidermis induced by HIV-1-infected cells depends on CCL5/RANTES.

### LCs are retained within the inner foreskin epidermis and capture HIV-1

LCs play a major role during the initial hours of HIV-1 exposure in both the male and female genital tracts, by sampling the mucosal surface and rapidly internalizing HIV-1 that remains intact within their cytoplasm [10], [25]. In the foreskin, LCs are located within the epidermis but not dermis [6]–[8], [10], [14], [15].

**Figure 4:**
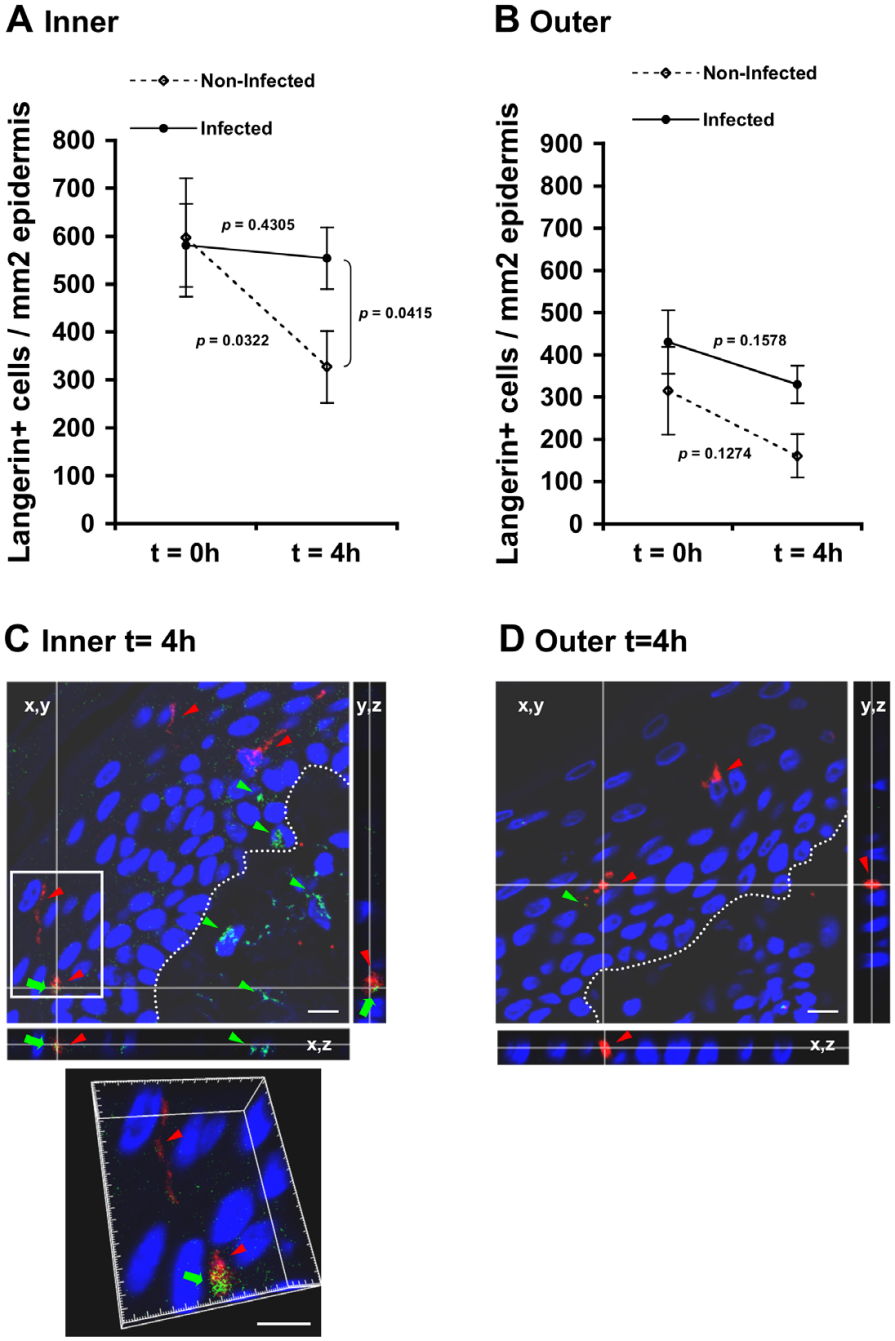
(A, B) HIV-1-infected cells induce retention of LCs within the epidermis. Parallel inner (A) and outer (B) foreskin explants were exposed for 4 h to either HIV-1-infected (solid lines) or non-infected (broken lines) PBMCs. Explants were then stained with anti-langerin Ab and visualized with DAB peroxidase substrate. Shown are means±SEM langerin+ cell densities (cells/mm^2^) in epidermis derived from three independent explants. Cells were counted in a minimum of 10 fields/section in each experiment. In (A): p = 0.0322 non-infected epidermis 4 h vs. 0 h and p = 0.0415 HIV-1-infected vs. non-infected at 4 h; Student's t-test. (C, D) HIV-1 entry and capture by LCs in inner, but not outer, foreskin explants. Single sections observed by confocal microscopy of parallel inner (C) and outer (D) foreskin explants, following 4 h exposure to HIV-1-infected PBMCs. Horizontal and vertical lines represent the localization of the sectioned stacks along the z axis (25 sections, 0.2 µm apart) that are shown below and to the right of each image. Sections were double-stained with goat-anti-human langerin Ab followed by TRITC-conjugated anti-goat IgG Ab and a mixture of several human/mouse anti-HIV-1 mAbs followed by FITC-conjugated anti-human/mouse IgG Abs. In (C), langerin is detected around cell bodies and dendrites reaching the apical surface (red arrowheads); HIV-1 virions are detected as dots in the epidermis associated with epithelial cells or in dermis (green arrowheads), as well as co-localized with LCs (green arrows). Higher magnification image with the xyz planes rotated shows viral particles internalized into LCs. Cell nuclei were counterstained with DAPI. White dotted lines denote the epidermis/dermis interface. Scale bars = 10 µm. Representative images of n = 3 experiments.

**Figure 5:**
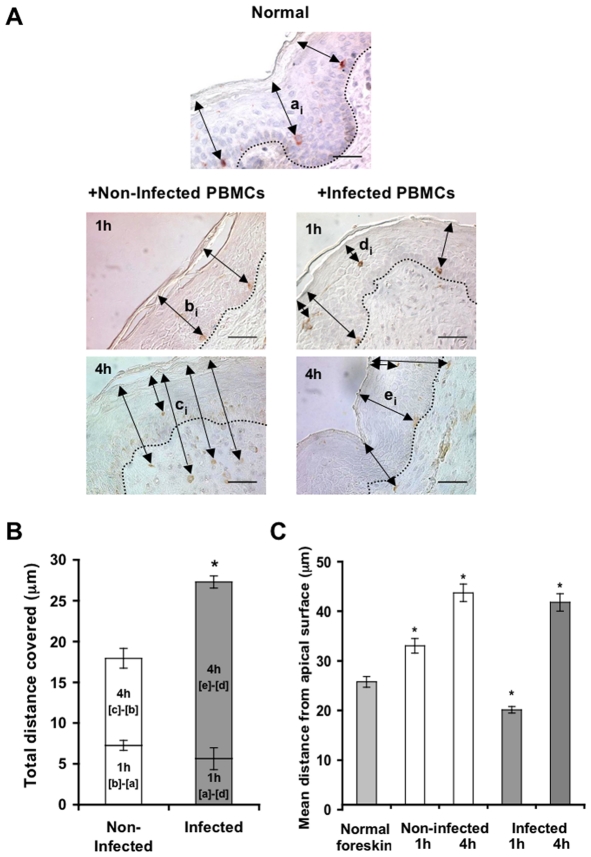
HIV-1-infected cells modify the spatial distribution of LCs in inner foreskin. (A) Representative images of normal inner foreskin (top), and inner foreskin explants exposed to either non-infected (left) or HIV-1-infected (right) PBMCs for 1 h (middle) or 4 h (bottom). Explants were then stained with anti-langerin Ab and visualized with AEC (normal foreskin) or DAB (foreskin explants) peroxidase substrates. The black double-headed arrows denote individual distances of LCs from the apical surface for each condition and are marked as [a_i_]-[e_i_] respectively. Images are representative of three independent experiments. Scale bars = 20 µm. (B) Calculated mean±SEM distances covered by LCs, following exposure of inner foreskin explants to either non-infected (white bars) or HIV-1-infected (grey bars) PBMCs. For exposure to non-infected PBMCs, the distances after 1 h and 4 h were calculated as [b]-[a] and [c]-[b], respectively, where a, b, c are means of the individual [a_i_], [b_i_], [c_i_] distances (measured for 106, 193 and 148 different cells in three independent explants). For exposure to HIV-1-infected PBMCs, the distances after 1 h and 4 h were calculated as [a]-[d] and [e]-[d], respectively, where d and e are mean of the individual [d_i_] and [e_i_] distances (measured for 186 and 108 different cells in three independent explants). *p<0.0001 infected vs. non-infected at 4 h; Student's t-test. (C) shows the actual distances from the apical surface measured for each experimental condition. *p<0.0001 vs. normal foreskin; Student's t-test.

To study possible changes in LC density mediated by HIV-1, inner and outer foreskin explants were inoculated for 4 h with either HIV-1-infected or non-infected PBMCs, stained for langerin, and the numbers of langerin+ LCs were counted. In the inner foreskin epidermis, LC density did not change upon exposure to HIV-1-infected cells (from 581±124 to 554±75; mean±SEM langerin+ cells/mm^2^ epidermis from n = 3 experiments; p = 0.4305, Student's t-test; Fig. 4A). In contrast, inoculation with non-infected PBMCs resulted in a significant decrease in LC density (from 597±87 to 327±64; mean±SEM langerin+ cells/mm^2^ epidermis from n = 3 experiments; p = 0.0322, Student's t-test; Fig. 4A), in correlation with detection of langerin+ cells in the dermis at this time point (see Fig. 5 below). The difference between the densities of epidermal LCs following exposure to HIV-1-infected compared to non-infected PBMCs at 4 h was significant (p = 0.0415, Student's t-test). No statistically significant changes were observed in the density of langerin+ cells in parallel outer foreskin explants exposed to either HIV-1-infected or non-infected PBMCs (p = 0.1578 and 0.1274, respectively, Student's t-test; Fig. 4B).

To investigate the co-localization of HIV-1 virions with LCs, inner and outer foreskin explants were exposed for 4 h to HIV-1-infected PBMCs, double stained for langerin and HIV-1, and examined by confocal microscopy. The specificity of the signals recorded was based on parametric settings using specific isotype controls for either langerin or HIV-1 [10]. In inner foreskin, langerin was detected around cell bodies, as well as in LC dendrites reaching the apical surface (Fig. 4C, red arrowheads). In these explants, HIV-1 was detected as free virions or associated with epithelial cells within the epidermis (Fig. 4C, green arrowheads). Notably, HIV-1 was also found co-localized with LCs (Fig. 4C, green arrows) including virions that were internalized into LCs (Fig. 4C, higher magnification insert), and was further detected within the dermis. In contrast, in outer foreskin, only few virions were detected within the epidermis (Fig. 4D), in agreement with our previous study [10].

These results suggest that HIV-1 originating from infected cells enters the inner foreskin, and is captured by LCs retained in the epidermis. In contrast, in outer foreskin, HIV-1 fails to enter the epidermis or affect LC density.

### HIV-1-infected cells modify the spatial distribution of LCs in the inner foreskin

To follow the localization of LCs following HIV-1 exposure, inner foreskin explants were exposed for 1 h or 4 h to either HIV-1-infected or non-infected PBMCs, stained for langerin, and the distances of each langerin+ cell from the apical surface (irrespective of their HIV-1 content) were measured. Similar distances were also measured in normal inner foreskin, serving as the set point for the spatial distribution of LCs.

Inoculation with non-infected PBMCs resulted in a time-dependent increase in the mean distance of LCs from the apical surface compared to normal inner foreskin (Fig. 5A). Thus, at 1 h LCs were localized closer to the basement membrane and at 4 h LCs crossed the epidermis/dermis interface and reached the dermis, localizing further away from the apical surface. In contrast, exposure to HIV-1-infected PBMCs resulted in a bi-phasic modification in LC spatial distribution, compared to normal foreskin (Fig. 5A): after 1 h of viral exposure, the mean distance of LCs from the apical surface shortened; at 4 hr these cells were localized near the basement membrane, and fewer LCs reached the dermis compared to exposure with non-infected PBMCs.

Based on the above ‘snap-shot’ images and to calculate the total distance covered by LCs during the 4 h infection period, the following mean distances from the apical surface were designated: [a] for normal tissue (set point distance); [b] and [c] after 1 h and 4 h inoculation with non-infected PBMCs, respectively; [d] and [e] after 1 h and 4 h inoculation with HIV-1-infected PBMCs, respectively (Fig. 5A). The mean distance covered by LCs following 1 h exposure to non-infected PBMCs is therefore represented by [b] minus [a], as LCs were localized away from the apical surface. In contrast, the mean distance covered by LCs following 1 h exposure to HIV-1-infected PBMCs is represented by [a]-[d], as LCs were localized closer to the apical surface. At 4 h, the additional distances covered by LCs are represented as [c]-[b] and [e]-[d], as LCs were localized away from the apical surface following exposure to either non-infected or HIV-1-infected PBMCs. This analysis (Fig. 5B, C) demonstrated that at 1 h post-infection, LCs covered a similar distance, but in opposite directions (7.259±0.606 µm basally and 5.642±1.337 µm apically after exposure to either non-infected or HIV-1-infected PBMCs, respectively). Yet, LCs covered a shorter additional distance at 4 h after exposure to non-infected, compared to HIV-1-infected PBMCs (10.677±1.218 µm and 21.651±0.749 µm for non-infected or HIV-1-infected PBMCs, respectively; p<0.0001, Student's t-test; Fig. 5C).

These findings suggest that LCs may directly exit the epidermis following inoculation with non-infected cells. However, HIV-1 originating from infected cells induces first the attraction of LCs to the apical surface at 1 h, followed by their entry deeper into the tissue towards the epidermis-dermis interface at 4 h.

### HIV-1-infected cells induces continuous formation of LC-T-cell conjugates

Following the rapid internalization of HIV-1 by LCs in the inner foreskin at 1 h, intact virions that are sequestered within LCs are transferred to epidermal T-cells across conjugates forming between LCs and T-cells [10]. The number of such LC-T-cell conjugates was further evaluated at 4 h, in inner foreskin explants inoculated with either HIV-1-infected or non-infected PBMCs and double stained for langerin and CD3. The percentage of langerin+/CD3+ conjugates out of the total number of langerin+ cells was then calculated. Of note, conjugates were defined as those in which LCs and T-cells were in direct contact with a visible interface and common segment of plasma membrane, while excluding those in which LCs and T-cells were only in proximity with a visible space between the two stained cells.

**Figure 6:**
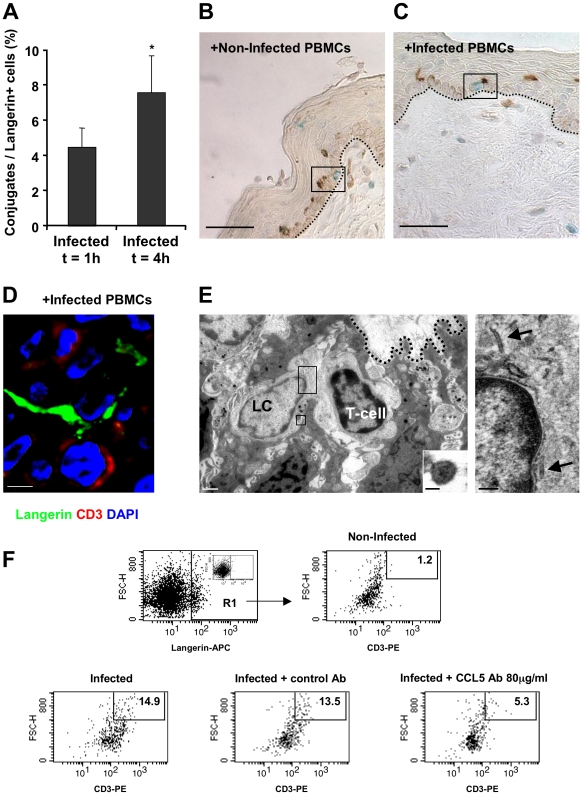
HIV-1-infected cells increase the formation of epidermal LC-T-cell conjugates. (A) Inner foreskin explants were exposed for 1 h or 4 h to HIV-1-infected PBMCs. Explants were then double stained with anti-langerin and anti-CD3 Abs, and visualized with DAB and HistoGreen peroxidase substrates, respectively. Shown are calculated means±SEM % LC-T-cell conjugates of the total LCs from three independent explants. Cells were counted in a minimum of 10 fields/section for each experiment. *p = 0.0431 4 h vs. 1 h, Student's t-test. (B–D) Representative images of inner foreskin explants exposed to either non-infected (B) or HIV-1-infected (C and D) PBMCs for 4 h, and double stained for langerin (DAB brown in B and C; Alexa488 green in D) and CD3 (Histogreen blue-green in B and C; Cy5 red in D). Single isolated non-conjugated LCs and T-cells are shown in the framed window in (B), while the framed window in (C) shows LC-T-cell conjugate at the epidermal-dermal interface above the basement membrane (black dotted lines). The confocal microscopy image in (D) shows the close contact between one LC and one T-cell, as well as the LC dendrite in proximity to a second T-cell. Scale bars = 20 µm (B, C) and 8 µm (D). (E) Electron micrograph of LC-T-cell conjugate in inner foreskin explant exposed for 4 h to HIV-1-infected PBMCs. The conjugate is positioned within the epidermis at the epidermal-dermal interface above the basement membrane (black dotted line); scale bar = 1 µm. Higher magnification of the top framed window (right side) shows two rod-shaped Birbeck granule in the LC cytoplasm (black arrows); scale bar = 200 nm. Higher magnification of the bottom framed window (right corner) shows an HIV-1 particle of 90 nm with a central dark core characteristic of mature virions associated with LC; scale bar = 50 nm. “(F) Inner foreskin explants were exposed for 4 h to either non-infected PBMCs (top right) or HIV-1-infected PBMCs: alone (bottom left); in the presence of 40 µg/ml control goat IgG Ab (bottom middle); in the presence of 80 µg/ml neutralizing goat Ab to CCL5/RANTES (bottom right). Following infection, epidermal single-cell suspensions were prepared and double stained with APC-conjugated anti-human langerin and PE-conjugated anti-human CD3 mAbs. Cells were first gated on langerin+ cells (R1 gate, top left profile, insert shows staining with matched isotype control). Numbers represent the percentages of cells in R1 that are both high forward scatter and CD3+. Images are representative of two independent experiments.

At 1 h, 4.5±1.1% LCs were conjugated with T-cells (Fig. 6A). The proportion of conjugates continued to increase and almost doubled at 4 h, with 7.6±2.1% of LCs forming conjugates with T-cells (p = 0.0431, Student's t-test, Fig. 6A). These LC-T-cell conjugates were located within the epidermis just above the basement membrane (Fig. 4C), while conjugates were only occasionally detected following exposure to non-infected PBMCs (Fig. 6B). Higher magnification images revealed LCs to be tightly associated with T-cells and HIV-1 virions (Fig. 6D, E).

In agreement with our conjugate counting by immunohistochemistry (see above), inoculation with HIV-1-infected PBMCs resulted in a higher percentage of langerin+/CD3+/high forward scatter conjugates in cell suspensions of inner foreskin epidermis, compared to inoculation with non-infected PBMCs (Fig. 6F). Pre-treatment of inner foreskin explants with a control isotype Ab did not affect the observed increase in conjugate formation upon exposure to HIV-1-infected cells. In sharp contrast, a neutralizing Ab to CCR5/RANTES inhibited the formation of LC-T-cell conjugates induced by HIV-1-infected PBMCs (Fig. 6F).

These results suggest that LC-T-cell conjugate formation within the inner foreskin tissue following exposure to HIV-1-infected cells is a sustained process during the early hours following exposure to HIV-1.

## Discussion

HIV-1 is a viral menace that gains access into the body mainly during sexual intercourse, by crossing epithelial barriers that cover the mucosal surfaces of the gastrointestinal, female and male genital tracts. In stratified epithelia, HIV-1 ‘highjacks’ the physiological process of pathogen recognition by LCs [26] in order to invade the body. In the inner foreskin [9], [10] and vagina [25], early HIV-1 transmission involves capture of HIV-1 by epidermal LCs. These cells are able to internalize intact HIV-1 virions, due to their close proximity to the mucosal surface and their ability to bind the HIV-1 envelope glycoprotein subunit gp120 via their unique C-type lectin langerin [19], [21], [22]. The internalized intact virus is then transferred to T-cells locally within the epithelium [10], [25]. Hence, LCs and T-cells are the first cells targeted by HIV-1 in both the male and female genital tracts early upon exposure to HIV-1.

To gain further insight as to the molecular/cellular dynamics of HIV-1 entry in the foreskin, we used in the current study our recently developed foreskin explants [10], which permit for polarized exposure to the virus only via the apical side, as takes place *in-vivo*. Foreskin explants were inoculated comparatively for 4 h with HIV-1-infected or non-infected cells, as our previous study clearly demonstrated that foreskin transmission of cell-associated HIV-1 is much more efficient compared to that of cell-free virus [10]. In line with these observations, a limited number of studies aimed at ascertaining the infectivity of cell-associated HIV-1 and its potential importance in mucosal transmission, reported that cell-associated HIV-1 was transmitted more efficiently compared to cell-free virus (e.g. [27], and recently reviewed in [28]).

To explore the possible molecular mechanisms that might affect the migratory behavior of T-cells and LCs in the foreskin, we measured the levels of several chemokines and cytokines that may be secreted by the inner foreskin tissue. Our results show that the inner foreskin produces high levels of CCL2/MCP-1, CXCL8/IL-8, IL-6 and CXCL10/IP-10, in line with a recent study that documented a similar patter of secretion in inner foreskin explants [16]. However, HIV-1-infected cells do no affect the secretion of these chemokines/cytokines (Fig. 1), suggesting that these molecules probably do not contribute to the observed modifications in the spatial distribution of T-cells/LCs reported herein that are mediated by HIV-1-infected cells. In contrast, HIV-1-infected cells up-regulate CCL5/RANTES and down-regulate CCL20/MIP-3 alpha secretion, during both the first and late hours following viral inoculation. We speculate that at the early time points, chemokine secretion represents the levels of pre-synthesized/stored chemokines, rather then *de-novo* synthesis. Hence, the exact mechanisms by which HIV-1-infected cells modify early/late chemokine secretion may differ.

Of note, the PHA/IL-2 activated PBMCs used herein may differ from naturally occurring HIV-1-infected cells in genital secretions and may secrete chemokines due to their experimental activation. Although both non-infected/HIV-1-infected cells secreted CCL5/RANTES and CCL20/MIP-3 alpha when incubated alone, without contact with the foreskin tissue, the secreted levels did not differ between non-infected and HIV-1-infected cells and were low (data not shown). In contrast, chemokines secretion was always higher in the supernatant fractions upon inoculation with the tissue explants, suggesting that the contact between HIV-1-infected cells and the inner foreskin tissue results in the observed modifications in chemokines secretion, which may be contributed by the cells themselves, the tissue, or both.

By measuring the density of T-cells in both the foreskin epidermis and dermis, we reveal herein that HIV-1-infected cell induce T-cell recruitment from the dermis into the epidermis, as the density of epidermal T-cell increases, in correlation with a decrease in their density in the dermis (Fig. 2). This process is restricted to the inner foreskin, as T-cell density remains unchanged in the outer foreskin, in line with previous studies including our own, showing that the inner, but not outer, foreskin is permissive to HIV-1 entry [7], [10]. Importantly, our studies clearly show that CCL5/RANTES, a known T-cell chemokine [23], [24], which is increased following inoculation with HIV-1-infected cells, is responsible for such T-cell recruitment, as an activity neutralizing Ab to CCL5/RANTES completely abrogates this process (Fig. 3). This principal finding is in line with a recent study showing that CCL5/RANTES mediates specific recruitment of T-cells in human skin xenografts *in-vivo* [29]. Another study reported that treatment of inner foreskin explants for several days with the chemokine CCL3/MIP-1 alpha, but not with several others, induces T-cell infiltration into the epidermis [30]. However, as we show herein, CCL3/MIP-1 alpha secretion is decreased in inner foreskin explants following exposure to HIV-1 at such late time points (Fig. 1), suggesting that virus-mediated recruitment of T-cell into the epidermis does not involve this chemokine. Interestingly, the CCL5/RANTES neutralizing Ab used herein not only prevents epidermal T-cell recruitment, but apparently also facilitates exit of T-cell from the epidermis (i.e. the fold value in Fig. 3B is 0.72±0.10 for 80 µg/ml neutralizing Ab). This observation suggests that endogenous tissue CCL5/RANTES has a physiological role in maintaining T-cells within the epidermis.

In addition, we show that HIV-1-infected cells do not affect the density of epidermal LCs (Fig. 4), but rather modify their spatial distribution (Fig. 5). Hence, HIV-1-infected cells first induce attraction of LCs to the apical surface, and only later LCs travel towards the basement membrane. In fact, during the first hour of viral exposure, LCs cover a similar distance, but in opposite directions: apically upon exposure to HIV-1-infected cells and basally upon exposure to non-infected cells. As measuring the levels of secreted chemokines after 1 h is technically challenging, the identity of the specific chemokines mediating these opposing effects is currently an open question. Interestingly, the additional migration distance covered by LCs later on after inoculation with HIV-1-infected cells is higher compared to non-infected cells (Fig. 5), suggesting that LCs are able to migrate more rapidly (i.e. longer distance during the same time period). This observation correlates with the decreased secretion levels of CCL20/MIP-3 alpha, the most potent chemokine for LCs [31], at this time point. In contrast, inoculation with non-infected cells results in a gradual increase in LC distance from the apical surface along with a decrease in their epidermal density. This process probably reflects the natural emigration of LCs out of the epidermis, a known feature of these migratory cells that is routinely used experimentally for their isolation form mucosal epithelia. We further speculate that HIV-1 might have evolved the ability to modulate chemokines secretion to mediate the initial attraction of LCs apically, in order to facilitate its capture by LCs. This notion is supported by our microscopy studies showing for the first time co-localization of HIV-1 with LCs *in-situ* at the early hours following viral exposure (Fig. 4). Such process could permit early local spread of the virus to epidermal T-cells (either already present within the epidermis and/or newly recruited from the dermis) and establishment of an HIV-1-infected founder cell population that is crucial for systemic dissemination of the virus later [32], [33].

Comparatively, in the female genital tract, HIV-1 enters lamina propria macrophages in explanted vaginal mucosa as early as 30 min after inoculation of virus onto the epithelium, and purified vaginal macrophages support substantial levels of HIV-1 replication [34]. In contrast, within the foreskin, dermal macrophages/DCs do not seem to participate in the early events of HIV-1 entry. Their densities and spatial distributions in the dermis remain completely unaffected by the presence of either HIV-1-infected or non-infected cells (data not shown). In addition, intact HIV-1 virions may enter vaginal epithelial cells [35], and also inner foreskin keratinocytes as shown here (Fig. 4). Whether these sequestered virions affect foreskin keratinocytes to promote viral entry is still unclear.

Productive infection of LCs with HIV-1 following viral capture is limited, and may rely on langerin-mediated degradation of low concentrations of HIV-1 [22] and expression of several host factors that block HIV-1 replication [36], [37]. In sharp contrast, previous studies have clearly demonstrated that HIV-1 captured by LCs can be efficiently transferred to T-cells across LC-T-cell conjugates, to induce extensive replication of the virus in T-cells [20], [22], [38]–[45]. In line with these studies, we recently reported that within 1 h, LCs in the inner foreskin internalize intact HIV-1 virions, which are transferred to T-cells across LC-T-cell conjugates forming rapidly within the epidermis [10]. In the female genital tract, endocytosed virions persist in LCs and remain accessible for transfer to conjugated vaginal T-cells [25]. Hence, LCs serve as storage for HIV-1, facilitating its later spread to T-cells.

Herein, we further followed the process of LC-T-cell conjugate formation in the inner foreskin at later time points. Such conjugated formation is initiated 1 h post HIV-1 inoculation and appears to be a continuous process with increase in conjugate numbers up to 4 h. Importantly, as LC density remains constant in the inner foreskin epidermis (Fig. 4), while T-cell density increases (Fig. 2), the recruitment of T-cell into the epidermis fuels this continuous and sustained formation of LC-T-cell conjugates. Thus, inhibiting the recruitment of T-cells into the epidermis may turn clinically useful to limit the local spread of the virus.

Clinically approved CCR5 inhibitors [46]–[48] prevent HIV-1 infection of CCR5-expressing cells, and are also efficient microbicides preventing vaginal transmission in female macaques [49], [50]. As demonstrated herein, CCL5/RANTES recruits T-cells into the inner foreskin epidermis, which drives the continuous process of LC-T-cell conjugate formation. Hence, CCR5 inhibitors may have a dual mechanism of action: directly inhibiting HIV-1 infection, as well as decreasing mucosal T-cell recruitment, to subsequently limit the local spread of the virus from LCs to T-cells. Future studies will hopefully challenge this notion, and examine the efficacy of CCR5 inhibitors as microbicides aimed at preventing inner foreskin HIV-1 entry.

**Figure 7:**
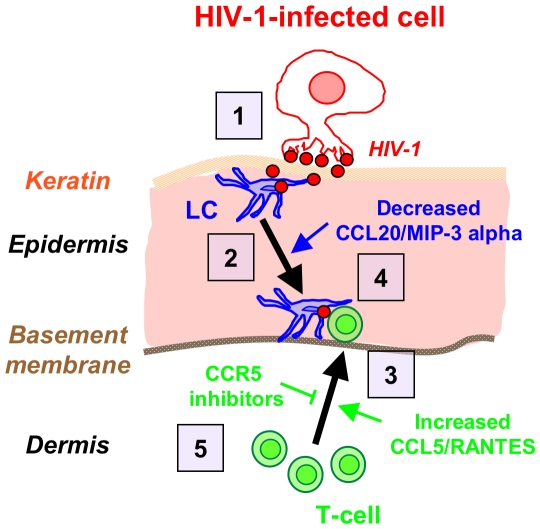
Schematized ‘chain-of-events’ of HIV-1 early entry in the inner foreskin. At 1 h, HIV-1-infected cells form apical viral synapses, which results in local HIV-1 budding, LCs attraction and HIV-1 capture (1). At 4 h, LCs migrate back towards the basement membrane deeper into the tissue (2), in correlation with reduced CCL20/MIP-3 alpha secretion. At this time point, T-cells are recruited from the dermis into the epidermis (3), as a result of increased CCL5/RANTES secretion, and fuel the formation of LC-T-cell conjugates (4). Blocking CCR5 on T-cells by clinically available CCR5 inhibitors may serve to limit T-cell recruitment and local viral spread (5).

Based on our results, Fig. 7 summarizes the ‘chain-of-events’ of early HIV-1 entry in the inner foreskin. At 1 h, the interaction of HIV-1-infected cells with the inner foreskin leads to viral synapse formation, HIV-1 budding and attraction of LCs to the apical surface, which facilitates viral capture by LCs. At 4 h, LCs harboring intact HIV-1 virions in their cytoplasm rapidly travel towards the basement membrane, and T-cells are recruited from the dermis into the epidermis, to fuel the continuous formation of LC-T-cell conjugates within the inner foreskin epidermis. The re-localization of LCs towards the basement membrane may result from decreased secretion of CCL20/MIP-3, while recruitment of T-cells results from increased CCL5/RANTES secretion, induced by HIV-1-infected cells. Blocking the responsiveness to these chemokines, for instance by CCR5 inhibitors already approved for clinical use, may turn useful as a novel mechanism to limit the local spread of HIV-1 within the inner foreskin epithelium, and perhaps also other mucosal epithelia.

## Materials and Methods

### Ethics statement

The study was performed according to local ethical regulations following approval by the local ethical committee (Comité de Protection des Personnes (GHU Cochin-St vincent de Paul, Paris, France). Written informed consent was provided by all study participants and/or their legal guardians.

### *Ex-vivo* polarized foreskin tissue explants

Normal foreskin tissues were obtained from the Urology Service at the Cochin Hospital, Paris, France, from healthy adults (mean age 32 years old, range 18–57 years) undergoing elective circumcision due to personal reasons or phimosis, and according to local ethical regulations. Foreskin tissues removed due to cancerous or infectious pathologies were discarded and not used. Informed consents were obtained from all individuals. Tissues were placed in phosphate-buffered saline (PBS) supplemented with 20 µg/ml gentamicin (Gibco Invitrogen, Carlsbad, CA), transported to the laboratory immediately following circumcision, and processed within the next 2 h.

Foreskin tissues were separated mechanically into inner and outer parts, distinguished by their different colors and morphology, and any remaining fat and muscle tissue was removed from the dermal side. Round pieces from either inner or outer foreskin were cut using a 8 mm Harris Uni-Core, and placed with their epidermal side facing up on top of a polycarbonate membrane (12 µm pore size, 12 mm diameter) of a two-chamber Costar Transwell permeable insert (Corning Inc, Corning, NY). Hollow plastic cloning ring cylinders of 6 mm inner diameter (VMR, Strasbourg, France) were glued to the apical surface of each tissue piece, using the two-component biological fibrin sealant Tissucol kit (Baxter Int., Vienna, Austria), as previously described [10]. Sealing efficiency of the polarized apical chambers was monitored as previously described [10].

### Virus and infected cells

PBMCs from healthy donors were separated from whole blood by a standard Ficoll gradient. The HIV-1 primary isolate 93BR029 (clade B, R5 tropic; NIH AIDS reagent program) was amplified on phytohaemagglutinin (PHA)-stimulated PBMCs as described [51]. HIV-1 p24 antigen was quantified by the p24 Innotest HIV-1 ELISA (Innogenetics, Gent, Belgium), and viral stocks were aliquoted for single use and stored at −80°c.

To obtain HIV-1-infected cells, PHA/IL-2 stimulated PBMCs (5×10^6^ cells in 10 ml RPMI 1640 (Gibco) supplemented with 10% fetal bovine serum (FBS; PAN Biotech GmbH, Aidenbach, Germany), 2 mM L-glutamine, 100 U/ml penicillin, 100 µg/ml streptomycin (Gibco), and 10 U/ml IL-2 (Roche Diagnostics GmbH, Mannheim, Germany)) were inoculated with 200 ng p24 of the HIV-1 primary isolate 93BR029. Two days later, further non-infected PHA/IL-2 stimulated cells were added (20×10^6^ cells in 10 ml of the same medium containing 20 U/ml IL-2). Infection was monitored in culture supernatants by p24 ELISA and by p24 intracellular staining and flow cytometry analysis. Infected cells were used between days 7–14 following addition of virus. Under these infection conditions, 1×10^6^ infected cells released 100–500 pg p24 after 1 hr incubation at 37°c, and 5–7% of the infected cells were positive for p24.

### Short-term polarized infection assay

Foreskin explants were exposed apically and in a polarized manner to 1×10^6^ of either HIV-1-infected or non-infected PBMCs (as negative control), in 100 µl RPMI 1640 added into the inner space of the cloning ring cylinders adhered to the tissue explants. Lower chambers were supplemented with 0.5 ml RPMI 1640. Following 4 h incubation at 37°c, explants were either fixed in 4% paraformaldehyde (PFA) for further morphological evaluation, or processed for preparation of epidermal single-cell suspensions (see below). Alternatively, following the 4 h polarized viral exposure, explants were washed and further incubated overnight at 37°c in a non-polarized manner in 0.5 ml RPMI 1640. For measurement of secreted chemokines and cytokines, the supernatants fractions were removed, centrifuged, inactivated at 56°c for 45 min, and stored at −80°c.

### Quantification of secreted chemokines and cytokines

The levels of eleven chemokines and cytokines, secreted by triplicate foreskin explants exposed to either HIV-1-infected/non-infected PBMCs (4 h polarized exposure followed by overnight non-polarized incubation), were measured by a custom multiplex bead immunoassay kit (Bender MedSystems, Vienna, Austria) and quantified by flow cytometry, according to the manufacturer's instructions. Recorded profiles from flow cytometry were exported and analyzed using the FlowCytomix Pro 2.3 Software (Bender) enabling calculation of the concentration (pg/ml) of each analyte in the tested samples. The levels of CCL20/MIP-3 alpha in supernatants collected after similar exposure to the virus, or following short-term polarized infection for 4 h, were quantified with the Quantikine human MIP-3 alpha ELISA kit (R&D systems, Minneapolis, MN), according to the manufacturer's instructions, and translated to actual concentrations (pg/ml) with the standard curve obtained using the kit standards. When indicated, CCL20/MIP-3 alpha and CCL5/RANTES levels following short-term polarized infection for 4 h were also evaluated by a custom semi-quantitative Human Cytokine Antibody Array and fluorescent detection (RayBiotech, Inc., Norcross, GA), according to the manufacturer's instructions.

### CCL5/RANTES blocking experiments in foreskin explants, epidermal single-cell suspensions and flow cytometry

Triplicate foreskin explants were pre-incubated for 30 min at 37°c with either 20 or 80 µg/ml of a goat-anti-human CCL5/RANTES neutralizing Ab (R&D), added to both apical and basal compartments. Normal goat IgG (Santa Cruz Biotechnology, Santa Cruz, CA) added at 40 µg/ml served as negative control. Explants were then inoculated with HIV-1-infected or non-infected PBMCs for 4 h as described above, and following removal of the cloning ring cylinders, pooled tissue pieces were incubated with their epidermal side facing up in 1 ml RPMI 1640 medium supplemented with 2.4 U/ml Dispase II (Roche Diagnostics GmbH, Mannheim, Germany) in a 12-wells plate overnight at 4°C. The epidermis and dermis were then mechanically separated using forceps. Epidermal single-cell suspensions were prepared by incubating pooled epidermal sheets from each triplicate in 1 ml 0.05% Trypsin/EDTA (Gibco) for 10 min at 37°C, followed by inactivation of trypsin with 1 ml FBS, mechanical disruption using a 10 ml pipette, filtration of released cells through a 40 µm nylon cell strainer and centrifugation.

For surface staining of CD3 and langerin, epidermal cell suspensions were resuspended in PBS and transferred to a 96 round-bottom wells plate (0.1–0.5×10^6^ cells/well). Cells were then incubated for 30 min on ice with 10 µg/ml of PE-conjugated mouse-anti-human CD3 (BD Pharmingen, San Jose, CA) and APC-conjugated mouse-anti-human langerin (R&D) mAbs diluted in PBS to a final volume of 50 µl/well. Cells were then washed, centrifuged, and fixed for 15 min at room temperature with 4% PFA. Cells stained with matched isotype control Abs served as negative control. Fluorescence profiles were recorded using a FACSCalibure and results were analyzed using the CellQuest Pro software.

### Confocal microscopy and immunohistochemistry

Following infection and fixation, foreskin explants were embedded in paraffin. Serial 4 µm sections were cut, deparaffinized in xylene and graded alcohol solutions, microwave heated in 10 mM citrate buffer pH = 6.0 for antigen retrieval, cooled down in the same buffer, and washed in PBS.

For confocal microscopy, sections were quenched with PBS/50 mM glycine/75 mM ammonium chloride (Sigma, St. Louis, MO) for 10 min at room temperature and blocked in PBS containing 20% horse serum (HS, Vector Laboratories, Burlingame, CA) and 0.1% Tween-20 (Sigma) for 1 h at room temperature. Following washes in PBS, sections were incubated overnight at 4°c with primary Abs diluted in PBS/2% HS/0.1% tween (50 µl/section), including goat-IgG-anti-human langerin at 20 µg/ml (R&D); mouse-IgG-anti-human CD3 at 10 µg/ml (DakoCytomation, Glostrup, Denmark); a cocktail of several mouse and human monoclonal Abs (5–10 µg/ml) recognizing HIV-1 gp41 (41A (Hybridolab, Pasteur Institute, Paris, France) 2F5, D50, 4E10 (NIH)), gp120 (2G12; NIH) and p24 (Kal-1; Dako). Sections were then washed with PBS and incubated for 1 h at room temperature with appropriate secondary Abs (50 µl/section, diluted 1∶50–1∶100 in PBS), including TRITC-conjugated anti-goat IgG (Beckman Coulter, Villepinte, France) and FITC-conjugated anti-human/mouse IgG (Jackson Immunoresearch, West Grove, PA), or Alexa488-conjugated anti-goat IgG (Invitrogen, Cergy Pontoise, France) and Cy5-conjugated anti-mouse IgG (Jackson). Cell nuclei were then stained with DAPI (Sigma; 50 µl/section, 10 min at room temperature), and the sections washed again in PBS and mounted with MOWIOL medium (Calbiochem Merck, Darmstadt, Germany), containing DABCO antifade (Sigma; 100 mg/ml). Staining was visualized with Leica TCS SP or DMI6000 microscopes (Leica Microsystems, Wetzlar, Germany). Signals were processed based on established parametric settings using specific isotype controls [10]. Acquired image stacks were analyzed with Imaris Software (Bitplane AG, Zurich, Switzerland). Image deconvolution processing was performed by the Maximal Likelihood Estimate procedure of the Huygens software (Scientific Volume Imaging BV, Hilversum, The Netherlands).

For immunohistochemistry, the VECTASTAIN Elite-ABC kit (Vector) was used according to the manufacturer's instructions. Sections were quenched with 3% hydrogen peroxide for 10 min at room temperature and blocked with PBS/3% HS for 20 min at room temperature. All Abs, including mouse-IgG2b-anti-human langerin (Abcam, Cambrige, UK) and mouse-IgG1-anti-human CD3 (Dako), as well as appropriate matching isotype controls, were diluted to 5–10 µg/ml in Ab diluent (Dako) and incubated with tissue sections for 30 min at room temperature (50 µl/section). After washing in PBS, sections were incubated with appropriate biotinylated secondary Abs followed by HRP-conjugated streptavidin (Vector; 30 µl/section, 20 min at room temperature), and visualized with di-amino benzidine (DAB; Dako), 3-amino-9-ethyl carbazole (AEC; Dako) or HistoGreen (AbCys, Paris, France) peroxidase substrates. Sections were counterstained for 30 sec with hematoxyline solution (Dako) and mounted with aqueous MOWIOL or non-aqueous Vectamount (Vector) media, according to the recommendations for each substrate. For double antigen staining a similar protocol was used with the addition of a quenching step using LinBlock (Abcys) between the first and second antigen staining. Images were taken using the ×40 objective of a Nikon E800 microscope (Nikon, Tokyo, Japan) equipped with a CCD QICAM camera (QImaging, Surrey, BC, Canada). For cell quantification, the ImageJ software (NIH) was used to count the number of positively stained cells in a minimum of 10 separate fields/stained section, and to calculate the surface of either the epidermis or dermis in each field. Measurement of the distance between 100–200 LCs and the mucosal surface was evaluated using the Arcgis V9.3 program (ESRI, Redlands, CA), as described earlier [10].

### Electron microscopy

Foreskin explants were fixed for 1 h with 3% glutaraldehyde. Samples were then postfixed in 1% osmium tetroxide in 0.1 M PBS and dehydrated in increasing graded alcochol solutions. After 10 min incubation in a mixture of 1∶2 epoxy propane and epoxy resin, the samples were embed in gelatine capsules with freshly prepared epoxy resin and polymerized at 60°c for 24 h. Sections (80 nm) were then cut with an ultramicrotome (Reichert Ultracut S, Reichert Technologies, Depew, NY), stained with uranyl acetate and Reynold's lead citrate, and observed with a transmission electron microscope (JEOL 1011, JEOL, Tokyo, Japan), equipped with a GATAN numerical camera (Gatan, Munich, Germany). Pictures were taken and digitalized with Digital Micrograph software (Gatan, Munich, Germany).

### Statistical analysis

Statistical significance was analyzed by the Student's t-test.

## References

[1] Auvert B, Taljaard D, Lagarde E, Sobngwi-Tambekou J, Sitta R (2005). Randomized, controlled intervention trial of male circumcision for reduction of HIV infection risk: the ANRS 1265 Trial.. PLoS Med.

[2] Bailey RC, Moses S, Parker CB, Agot K, Maclean I (2007). Male circumcision for HIV prevention in young men in Kisumu, Kenya: a randomised controlled trial.. Lancet.

[3] Gray RH, Kigozi G, Serwadda D, Makumbi F, Watya S (2007). Male circumcision for HIV prevention in men in Rakai, Uganda: a randomised trial.. Lancet.

[4] Quinn TC (2007). Circumcision and HIV transmission.. Curr Opin Infect Dis.

[5] Ross HM, Romrell LJ, Kaye GI *Histology, a text an atlas*.. Third ed ed, ed. P.A. Coryell.

[6] Hussain LA, Lehner T (1995). Comparative investigation of Langerhans' cells and potential receptors for HIV in oral, genitourinary and rectal epithelia.. Immunology.

[7] Patterson BK, Landay A, Siegel JN, Flener Z, Pessis D (2002). Susceptibility to human immunodeficiency virus-1 infection of human foreskin and cervical tissue grown in explant culture.. Am J Pathol.

[8] McCoombe SG, Short RV (2006). Potential HIV-1 target cells in the human penis.. Aids.

[9] Ganor Y, Bomsel M (2010). HIV-1 Transmission in the Male Genital Tract.. Am J Reprod Immunol.

[10] Ganor Y, Zhou Z, Tudor D, Schmitt A, Vacher-Lavenu MC (2010). Within 1 h, HIV-1 uses viral synapses to enter efficiently the inner, but not outer, foreskin mucosa and engages Langerhans-T cell conjugates.. Mucosal Immunol.

[11] Qin Q, Zheng XY, Wang YY, Shen HF, Sun F (2009). Langerhans' cell density and degree of keratinization in foreskins of Chinese preschool boys and adults.. Int Urol Nephrol.

[12] Dinh MH, McRaven MD, Kelley Z, Penugonda S, Hope TJ (2010). Keratinization of the adult male foreskin and implications for male circumcision.. Aids.

[13] Pask AJ, McInnes KJ, Webb DR, Short RV (2008). Topical oestrogen keratinises the human foreskin and may help prevent HIV infection.. PLoS ONE.

[14] Donoval BA, Landay AL, Moses S, Agot K, Ndinya-Achola JO (2006). HIV-1 target cells in foreskins of African men with varying histories of sexually transmitted infections.. Am J Clin Pathol.

[15] Hirbod T, Bailey RC, Agot K, Moses S, Ndinya-Achola J (2010). Abundant expression of HIV target cells and C-type lectin receptors in the foreskin tissue of young Kenyan men.. Am J Pathol.

[16] Fischetti L, Barry SM, Hope TJ, Shattock RJ (2009). HIV-1 infection of human penile explant tissue and protection by candidate microbicides.. Aids.

[17] Soilleux EJ, Coleman N (2004). Expression of DC-SIGN in human foreskin may facilitate sexual transmission of HIV.. J Clin Pathol.

[18] Valladeau J, Ravel O, Dezutter-Dambuyant C, Moore K, Kleijmeer M (2000). Langerin, a novel C-type lectin specific to Langerhans cells, is an endocytic receptor that induces the formation of Birbeck granules.. Immunity.

[19] Turville SG, Cameron PU, Handley A, Lin G, Pohlmann S (2002). Diversity of receptors binding HIV on dendritic cell subsets.. Nat Immunol.

[20] Geijtenbeek TB, Kwon DS, Torensma R, van Vliet SJ, van Duijnhoven GC (2000). DC-SIGN, a dendritic cell-specific HIV-1-binding protein that enhances trans-infection of T cells.. Cell.

[21] Figdor CG, van Kooyk Y, Adema GJ (2002). C-type lectin receptors on dendritic cells and Langerhans cells.. Nat Rev Immunol.

[22] de Witte L, Nabatov A, Pion M, Fluitsma D, de Jong MA (2007). Langerin is a natural barrier to HIV-1 transmission by Langerhans cells.. Nat Med.

[23] Schall TJ, Bacon K, Toy KJ, Goeddel DV (1990). Selective attraction of monocytes and T lymphocytes of the memory phenotype by cytokine RANTES.. Nature.

[24] Schall TJ (1991). Biology of the RANTES/SIS cytokine family.. Cytokine.

[25] Hladik F, Sakchalathorn P, Ballweber L, Lentz G, Fialkow M (2007). Initial events in establishing vaginal entry and infection by human immunodeficiency virus type-1.. Immunity.

[26] Merad M, Ginhoux F, Collin M (2008). Origin, homeostasis and function of Langerhans cells and other langerin-expressing dendritic cells.. Nat Rev Immunol.

[27] Salle B, Brochard P, Bourry O, Mannioui A, Andrieu T (2010). Infection of macaques after vaginal exposure to cell-associated simian immunodeficiency virus.. J Infect Dis.

[28] Anderson DJ, Politch JA, Nadolski AM, Blaskewicz CD, Pudney J (2010). Targeting Trojan Horse leukocytes for HIV prevention.. Aids.

[29] de Nadai P, Chenivesse C, Gilet J, Porte H, Vorng H (2006). CCR5 usage by CCL5 induces a selective leukocyte recruitment in human skin xenografts in vivo.. J Invest Dermatol.

[30] Fahrbach KM, Barry SM, Anderson MR, Hope TJ (2010). Enhanced cellular responses and environmental sampling within inner foreskin explants: implications for the foreskin's role in HIV transmission.. Mucosal Immunol.

[31] Dieu-Nosjean MC, Massacrier C, Homey B, Vanbervliet B, Pin JJ (2000). Macrophage inflammatory protein 3alpha is expressed at inflamed epithelial surfaces and is the most potent chemokine known in attracting Langerhans cell precursors.. J Exp Med.

[32] Haase AT (2010). Targeting early infection to prevent HIV-1 mucosal transmission.. Nature.

[33] Li Q, Estes JD, Schlievert PM, Duan L, Brosnahan AJ (2009). Glycerol monolaurate prevents mucosal SIV transmission.. Nature.

[34] Shen R, Richter HE, Clements RH, Novak L, Huff K (2009). Macrophages in vaginal but not intestinal mucosa are monocyte-like and permissive to human immunodeficiency virus type 1 infection.. J Virol.

[35] Hladik F, McElrath MJ (2008). Setting the stage: host invasion by HIV.. Nat Rev Immunol.

[36] Piguet V, Steinman RM (2007). The interaction of HIV with dendritic cells: outcomes and pathways.. Trends Immunol.

[37] Wu L, KewalRamani VN (2006). Dendritic-cell interactions with HIV: infection and viral dissemination.. Nat Rev Immunol.

[38] de Jong MA, de Witte L, Oudhoff MJ, Gringhuis SI, Gallay P (2008). TNF-alpha and TLR agonists increase susceptibility to HIV-1 transmission by human Langerhans cells ex vivo.. J Clin Invest.

[39] Fahrbach KM, Barry SM, Ayehunie S, Lamore S, Klausner M (2007). Activated CD34-Derived Langerhans Cells Mediate Transinfection with Human Immunodeficiency Virus.. J Virol.

[40] Kawamura T, Cohen SS, Borris DL, Aquilino EA, Glushakova S (2000). Candidate microbicides block HIV-1 infection of human immature Langerhans cells within epithelial tissue explants.. J Exp Med.

[41] Kawamura T, Koyanagi Y, Nakamura Y, Ogawa Y, Yamashita A (2008). Significant virus replication in Langerhans cells following application of HIV to abraded skin: relevance to occupational transmission of HIV.. J Immunol.

[42] Pope M, Betjes MG, Romani N, Hirmand H, Cameron PU (1994). Conjugates of dendritic cells and memory T lymphocytes from skin facilitate productive infection with HIV-1.. Cell.

[43] Sugaya M, Lore K, Koup RA, Douek DC, Blauvelt A (2004). HIV-infected Langerhans cells preferentially transmit virus to proliferating autologous CD4+ memory T cells located within Langerhans cell-T cell clusters.. J Immunol.

[44] Reece JC, Handley AJ, Anstee EJ, Morrison WA, Crowe SM (1998). HIV-1 selection by epidermal dendritic cells during transmission across human skin.. J Exp Med.

[45] Cameron PU, Freudenthal PS, Barker JM, Gezelter S, Inaba K (1992). Dendritic cells exposed to human immunodeficiency virus type-1 transmit a vigorous cytopathic infection to CD4+ T cells.. Science.

[46] Lusso P (2006). HIV and the chemokine system: 10 years later.. Embo J.

[47] Kuritzkes DR (2009). HIV-1 entry inhibitors: an overview.. Curr Opin HIV AIDS.

[48] Biswas P, Tambussi G, Lazzarin A (2007). Access denied? The status of co-receptor inhibition to counter HIV entry.. Expert Opin Pharmacother.

[49] Veazey RS, Ketas TJ, Dufour J, Moroney-Rasmussen T, Green LC (2010). Protection of rhesus macaques from vaginal infection by vaginally delivered maraviroc, an inhibitor of HIV-1 entry via the CCR5 co-receptor.. J Infect Dis.

[50] Lederman MM, Veazey RS, Offord R, Mosier DE, Dufour J (2004). Prevention of vaginal SHIV transmission in rhesus macaques through inhibition of CCR5.. Science.

[51] Lagaye S, Derrien M, Menu E, Coito C, Tresoldi E (2001). Cell-to-cell contact results in a selective translocation of maternal human immunodeficiency virus type 1 quasispecies across a trophoblastic barrier by both transcytosis and infection.. J Virol.

